# Recurrent Thromboembolic Events While on Anticoagulation Lead to the Diagnosis of Metastatic Lung Adenocarcinoma: A Case Report

**DOI:** 10.7759/cureus.37827

**Published:** 2023-04-19

**Authors:** Heather A Brubaker, Damian H Sooklal

**Affiliations:** 1 Internal Medicine, Edward Via College of Osteopathic Medicine, Blacksburg, USA; 2 Internal Medicine, Johnston Memorial Hospital, Abingdon, USA

**Keywords:** hypercoagulable state, ischemic colitis, pulmonary emboli, metastatic adenocarcinoma of lung, thromboembolic events

## Abstract

Lung cancer can lead to hypercoagulability that causes thromboembolic events such as pulmonary emboli, deep vein thrombosis, ischemic strokes, and non-bacterial thrombotic endocarditis. While it is not uncommon for cancer to cause thromboembolic events, it is unusual for thrombotic events to be the first manifestation of cancer. In the following report, we review the case of a 59-year-old woman who presented with melena and abdominal pain. She had a pertinent history of multiple thromboembolisms while on anticoagulation four months before this presentation. Upon admission, it was discovered that the patient had new pulmonary emboli, and further workup revealed that her gastrointestinal symptoms were due to ischemic colitis. While initial imaging showed no evident masses that would heighten suspicion of cancer, she had persistent abdominal lymphadenopathy. Therefore, she also underwent an abdominal lymph node biopsy which revealed metastatic lung adenocarcinoma, a possible cause of her hypercoagulable state. This case report highlights the importance of considering malignancy in the differential of a patient with recurrent thromboembolism and raises the question of whether standardized screening for malignancy in patients with multiple thromboembolic events would be beneficial.

## Introduction

As is true with most neoplasms, lung cancer can have a variety of adverse effects on the body, including hypercoagulability, which can lead to thromboembolic events such as pulmonary emboli (PE), deep vein thrombosis (DVT), ischemic strokes, and non-bacterial thrombotic endocarditis (NBTE) [1-3]. Hypercoagulability due to cancer is, in part, due to an imbalance between factors that drive coagulation and fibrinolysis [4]. Cancer cells can release procoagulant factors such as tissue factor and thrombin, and there is often inflammation leading to the recruitment of proinflammatory cytokines such as tumor necrosis factor-α (TNF-α) and interleukin-1 (IL-1) [4,5]. The production of these factors leads to a hypercoagulable state which promotes coagulation and thrombi formation [2].

NBTE is a rare complication associated with a hypercoagulable state and occurs when thrombi form on the valves of the heart [6]. These thrombi can break off and travel through the bloodstream, potentially blocking blood flow to other organs, such as the intestine, which can lead to ischemic colitis [7,8]. Ischemic colitis is a condition in which the intestine becomes inflamed due to a lack of blood flow which can be caused by vaso-occlusive microthrombi [9]. This leads to presenting symptoms such as abdominal pain, diarrhea, melena, and hematochezia and can be diagnosed via colonoscopy [9,10]. Ischemic colitis is rarely seen secondary to a hypercoagulable state from an underlying malignancy [11].

While thromboembolic events are a rare presentation of malignancy, they may precede the diagnosis of cancer more often than we previously realized. For instance, a prospective follow-up study by Oudega et al. discovered that in a two-year follow-up period, 4.4% of all DVT patients were diagnosed with an underlying malignancy [12]. Nonetheless, it is still considered uncommon for cancer to present with venous thromboembolism (VTE), with one study reporting only 0.11% of patients with a cancer diagnosis having an associated VTE before diagnosis [13]. In the case of a middle-aged female, multiple thromboembolic events while on anticoagulation led to the eventual diagnosis of underlying stage IV metastatic adenocarcinoma of lung origin. This case demonstrates a rare presentation of lung adenocarcinoma and highlights the importance of considering underlying malignancy in the differential of a patient with recurrent unexplained thromboembolic events.

## Case presentation

This case focuses on a 59-year-old Caucasian female with an extensive medical history, including cerebrovascular accident, splenic infarcts, non-ST-elevation myocardial infarction, and culture-negative endocarditis, which all occurred two months prior. Her other medical problems included chronic abdominal pain, deep vein thromboses while on anticoagulation, gastroesophageal reflux disorder, hyperlipidemia, hypertension, and hypothyroidism. She was also a former smoker, cessation having occurred two months prior, with a history of one pack per day for 35 years. The patient presented to the emergency department (ED) with melena and uncontrolled abdominal pain on oxycodone-acetaminophen 5-325 mg every eight hours as needed, prescribed three days prior. She had three episodes of black stool in the past day and an episode of lightheadedness after the last bowel movement. She reported intermittent lightheadedness for the past three weeks without syncope. She had diffuse, non-radiating abdominal pain rated 10/10 on the pain scale with no alleviating factors and had no accompanying nausea, vomiting, or hematemesis. At the time, she was on enoxaparin 70 mg twice daily for anticoagulation. Upon arrival at the ED, she was hypotensive with a blood pressure of 82/54. Physical examination was benign, apart from generalized abdomen tenderness with no rebound or guarding. Due to her hypotensive state, she was given a 1,000 mL normal saline bolus; however, her blood pressure remained low, and she required an additional 1,000 mL bolus.

**Figure 1:**
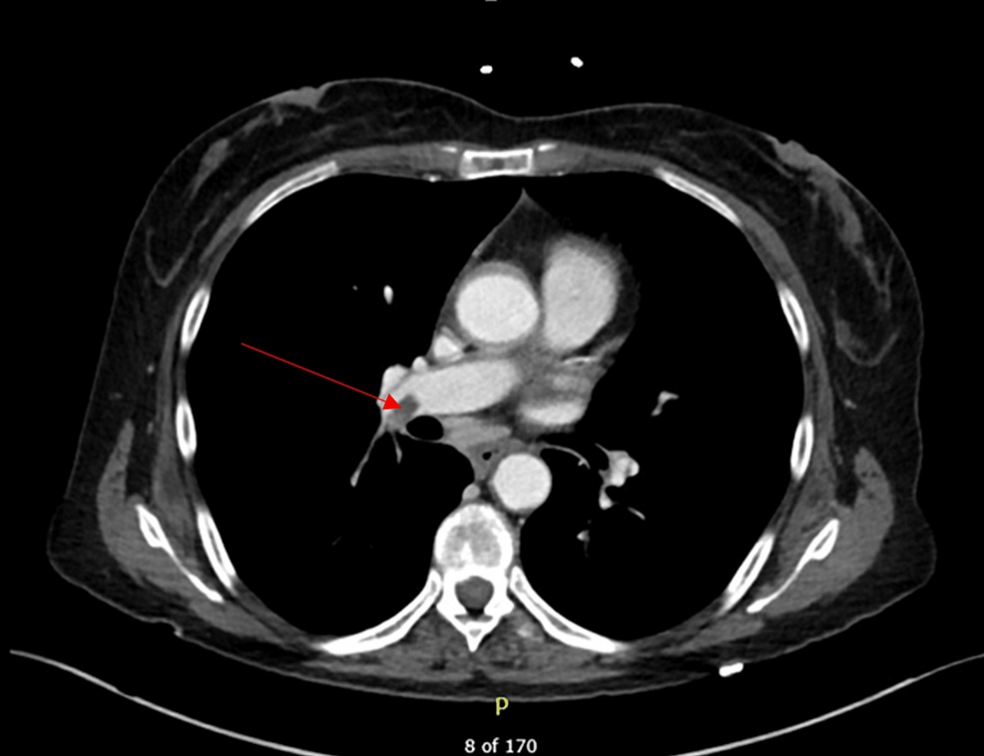
Right-sided pulmonary embolism (indicated by arrow) visualized on CT scan upon patient presentation to the ED

Based on the patient’s presentation, it was suspected that she had a gastrointestinal (GI) bleed with a differential of diverticulitis, colitis, diverticulosis, or GI mass. Studies were obtained, including complete blood count (CBC), comprehensive metabolic panel (CMP), troponin, chest x-ray, and CT abdomen/pelvis. There was leukocytosis of 18.6 K/µL, and hemoglobin was borderline elevated at 15.3 g/dL. Due to the leukocytosis, she received one dose of piperacillin-tazobactam 3.375 g via IV to treat possible infection. Blood cultures and urinalysis were also ordered. CT abdomen/pelvic scan showed mild colonic wall thickening involving the transverse colon and descending colon, suggesting possible colitis and new right upper and lower lobe pulmonary emboli without cardiac strain (Figure 1). The patient has a history of DVTs, pulmonary embolism, and abdominal pain in the four months leading up to this admission, for which she had been in and out of the hospital. On the CT abdomen/pelvic, para-aortic, pericaval, periceliac, and peri-superior mesenteric artery (SMA) lymphadenopathy was also appreciated and consistent with previous CT scans over the past three months. The patient had an abdominal lymph node biopsy scheduled for the following week to further investigate the persistent abdominal lymphadenopathy.

Gastroenterology was consulted and recommended starting a heparin drip for pulmonary emboli and pantoprazole 40 mg twice daily for suspected GI bleeding with possible esophagogastroduodenoscopy (EGD) and colonoscopy. The patient was admitted to the progressive care unit (PCU) for further workup and monitoring. Additional labs were ordered, including lactic acid within normal limits at 1.1 mmol/L, a borderline elevated C-reactive protein (CRP) of 10.4 mg/L, a negative urinalysis, a positive hemoccult, and a borderline elevated troponin at 0.04 ng/mL. Chest pain resolved, and repeat troponins down trended to 0.03 ng/mL and were discontinued. Upon transfer to the PCU, the patient was placed on a normal saline drip at 100 mL/hour for blood pressure support. Her antibiotics were changed from piperacillin-tazobactam to ceftriaxone 1 g daily and metronidazole 500 mg twice daily for suspected colitis. She was started on hydrocodone-acetaminophen 7.5-325 mg and hydromorphone 0.5 mg as needed for her abdominal pain. Hemoglobin and hematocrit were monitored every six to eight hours, and over the next 24 hours, her hemoglobin dropped from 15.3 g/dL to 12.0 g/dL to 10.3 g/dL, during which she remained on a heparin drip. The patient had continued diarrhea, melena, and abdominal pain for the next three days after admission, which was only mildly improved with hydrocodone-acetaminophen and hydromorphone. At this time, her leukocyte count improved to 8.4 K/µL which was within normal limits. On day three of admission, she underwent EGD and colonoscopy, revealing esophagitis, gastritis, duodenitis, sigmoid diverticulosis, a non-bleeding internal hemorrhoid, and most concerningly a wide linear ulceration worst at the splenic flexure and distal sigmoid which was concerning for ischemic colitis. Antibiotics were discontinued following the EGD and colonoscopy as stool cultures and three-day preliminary blood cultures were negative, ruling out an infectious cause. Following EGD and colonoscopy, the patient’s melena and diarrhea resolved; however, her abdominal pain persisted. Dicyclomine 10 mg three times daily was added to her pain management regimen to improve abdominal cramping.

**Figure 2:**
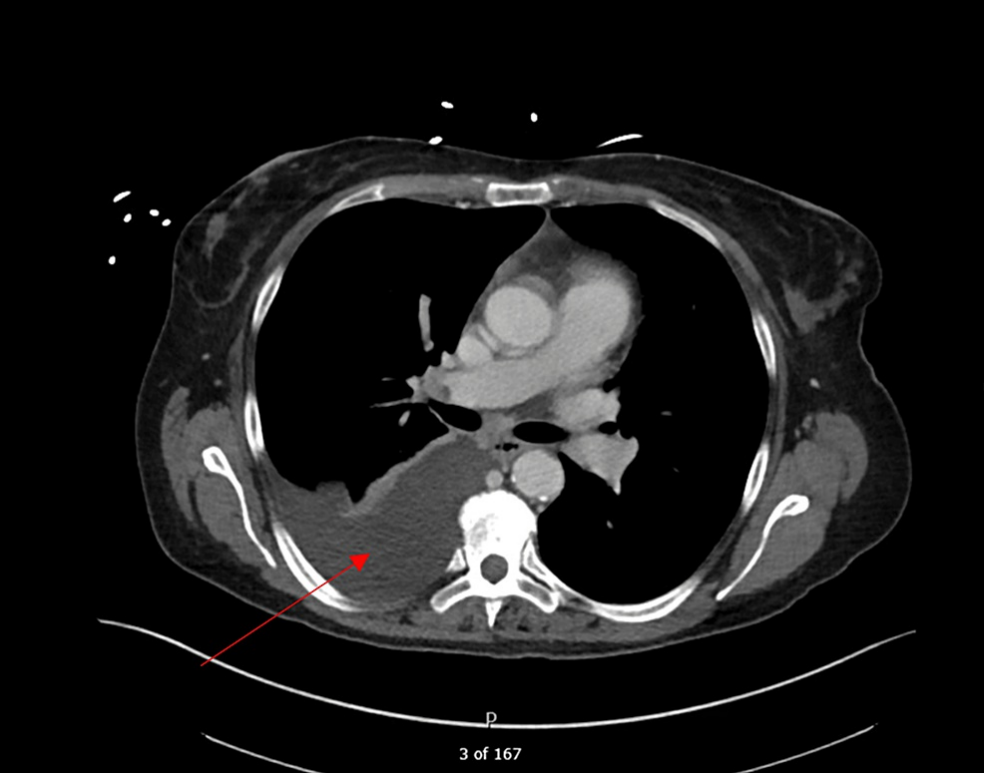
Moderate right pleural effusion (indicated by arrow) visualized on CT scan upon patient developing right-sided basilar crackles on day five of her admission

On day five of admission, the patient underwent an abdominal lymph node biopsy. Before the procedure, her heparin drip was held, and afterward, her anticoagulation regimen was switched to enoxaparin 80 mg twice daily. Because the patient was in stable condition, she was transferred from the PCU to the medical/surgical floor to await biopsy results. Following the procedure, the patient experienced anuria and was diagnosed with acute urinary retention that resolved with Foley catheterization. She was placed on nasal cannula oxygen (3L) and then developed new right-sided basilar crackles. A CT scan showed moderate right-sided pleural effusion (Figure 2). The pleural effusion was managed conservatively with incentive spirometry and continued nasal cannula oxygen.

On day eight of admission, the abdominal lymph node biopsy results showed metastatic adenocarcinoma, believed to be of lung origin. Immunohistochemistry was positive for cytokeratin 7 (CK7), thyroid transcription factor 1 (TTF1), and P16 and was negative for CK20, caudal type homeobox transcription factor 2 (CDX2), special AT-rich sequence-binding protein 2 (SATB2), and GATA. Upon receiving these results, an outpatient appointment with hematology and oncology was scheduled for the following week to determine a treatment approach. The patient was still experiencing significant abdominal pain, worse on the left, which was uncontrolled with her current pain regimen of IV hydromorphone 0.5 mg every four hours as needed, hydrocodone-acetaminophen 10-325 mg every six hours as needed, and dicyclomine 10 mg three times daily. Therefore, her regimen was changed to hydromorphone patient-controlled analgesia. Palliative care was consulted the following day to make a plan to transition the patient to a manageable outpatient regimen, including a fentanyl patch of 100 µg/hr with oxycodone 20 mg every four hours as needed. After being discharged, the patient followed up with hematology and oncology for further treatment and management of her stage IV metastatic lung adenocarcinoma.

## Discussion

While it is known that lung cancer is associated with hypercoagulability and thromboembolism, it is uncommon for thromboembolism to be the presenting sign of lung cancer [1-3,13]. This report reviews the case of a 59-year-old Caucasian woman who experienced recurrent thromboembolic events while on anticoagulation with enoxaparin before being diagnosed with underlying metastatic lung adenocarcinoma. One of the challenges that contributed to the delay in diagnosing this patient included the absence of a primary lesion. The patient underwent various imaging studies, including chest x-rays and abdomen/pelvic CT scans, in the four months before her diagnosis. However, there was no identifiable lesion suggesting a primary tumor that would warrant further investigation. Although the absence of a primary lesion contributed to the difficulty in diagnosing this patient, her presentation of abdominal pain further confounded efforts to identify her underlying cancer. While her abdominal pain was accompanied by lymphadenopathy, it was initially suspected to be reactive lymphadenopathy. Therefore, a biopsy, which would lead to her eventual diagnosis, was not performed for several months. Additional investigation of her melena and abdominal pain ultimately led to the diagnosis of ischemic colitis. It was hypothesized that her symptoms were secondary to her previously identified culture-negative endocarditis, likely NBTE. The etiology of her ischemic colitis is most likely due to the showering of thrombi from her heart to her bowel, leading to vaso-occlusion and her gastrointestinal symptoms. This presentation is rare and consistent with her recent history of recurrent thromboembolic events.

While it appears that thromboembolic events rarely precede a cancer diagnosis, this case report and other studies, such as the one conducted by Oudega et al., suggest that thromboembolisms preceding cancer diagnosis may be more prevalent than previously realized [12]. This finding raises the question of whether screening patients with venous thromboembolism for underlying cancer would be beneficial. Multiple studies investigated screening protocols regarding unprovoked VTEs and looked at limited screening (blood count, kidney function, ionogram, and chest x-ray) versus extended screening (ultrasound, CT, positron emission tomography (PET)/CT, endoscopy) [14,15]. In the study conducted by Ferreira et al., they developed a protocol that looks at patient risk factors, clinical history, physical examination, general laboratory results, imaging results, and Registro Informatizado Enfermedad TromboEmbólica (RIETE) score to determine if a patient should undergo limited or extended screening following unprovoked VTE [15]. While this approach may aid in the earlier identification of patients with cancer, it may not detect cancer in the absence of a primary lesion unless a PET scan is utilized in the screening process. Therefore, the benefits and drawbacks of screening, such as cost and radiation exposure, would need to be further investigated, as suggested in previous studies [14,15].

Overall, this patient’s presentation was genuinely concerning and should have raised suspicion of underlying carcinoma due to the numerous thromboembolic events that she experienced while on anticoagulation therapy. With her diagnosis of metastatic lung adenocarcinoma, she was started on a systemic chemotherapy regimen, including agents carboplatin with paclitaxel three times a week every four weeks and pembrolizumab on the first day of each cycle. This treatment regimen is consistent with the recommended treatment of metastatic lung adenocarcinoma [16]. In addition to her chemotherapy, she continued a pain management regimen and underwent other palliative treatments to preserve her quality of life. Unfortunately, the prognosis for stage IV lung adenocarcinoma is poor, with a two-year survival rate of 10%-15% [17]. The poor prognosis of advanced-stage lung cancer and the heightened prevalence of lung cancer in smokers are the reasons why it is recommended that high-risk individuals undergo low-dose CT screening [18]. While a low-dose CT screening likely would not have benefited this patient, her outcome might have been more favorable if she had received a diagnosis earlier during her presentation. This case highlights how important it is for clinicians to maintain a high level of clinical suspicion for underlying malignancy when treating patients with recurrent thromboembolic events.

## Conclusions

Cancer is associated with increased hypercoagulability, which can lead to thromboembolisms that may be the presenting sign of an underlying malignancy. Therefore, physicians must consider malignancy in the differential of patients with recurrent thromboembolic events. Although it appears uncommon for malignancy to present as venous thromboembolism, it may be more frequent than previously realized. This insight raises the question of whether screening for malignancy in patients with VTE would provide improved detection and thus allow for a better prognosis for patients with cancer. While further research will be necessary to assess whether screening protocols will be beneficial, the authors hope this report encourages the investigation of the risks, benefits, and costs of screening for malignancy in patients with multiple thromboembolic events.
